# T cell engagers: expanding horizons in oncology and beyond

**DOI:** 10.1038/s41416-025-03125-y

**Published:** 2025-07-23

**Authors:** Gulsah Albayrak, Peter Kok-Ting Wan, Kerry Fisher, Leonard W. Seymour

**Affiliations:** 1 Department of Oncology, University of Oxford, Oxford, UK; 2 Centre for Immuno-Oncology, Nuffield Department of Medicine, University of Oxford, Oxford, UK

**Keywords:** Cancer microenvironment, Immunization

## Abstract

**Background/introduction:**

T cell engagers (TCEs) are engineered immunotherapeutic molecules designed to direct the body’s immune system against tumour or infected cells by bridging T cells and their targets, triggering potent cytotoxic responses. Over the past decade, TCE-based therapies have gained momentum in oncology, resulting in several FDA approvals for haematologic malignancies and showing growing promise in solid tumours.

**Objective:**

This review elaborates on TCE mechanisms of action, emphasising their ability to activate T cells, target tumour antigens, and modulate the tumour microenvironment.

**Methods/results:**

We also delve into the clinical outcomes demonstrating TCE efficacy, alongside challenges such as cytokine release syndrome, antigen heterogeneity, and short half-lives. Recent advances in TCE design have incorporated multispecific constructs and conditional activation strategies and expansion in target molecules has enabled broadening applications to non-oncology indications like autoimmune and infectious diseases. Moreover, the use of artificial intelligence (AI) has also accelerated TCE discovery by identifying favourable epitope interactions, reducing immunogenicity risks, and enhancing overall design efficiency.

**Conclusions:**

Looking further, these advances open up a new era to redefine success for TCEs in both cancer and beyond, offering hope for more effective, safer immunotherapies.

## Introduction

The introduction of T cell engagers (TCEs) represented a major breakthrough in immunotherapy, offering a new method to activate T cells for the targeted destruction of cancer cells. In the evolving landscape of cancer immunotherapy, TCEs provided a pivotal innovation, harnessing the body's immune system to target and eliminate cancer cells. TCEs are a class of bispecific antibodies currently under clinical trials in different malignancies [1].

The approval of the CD19/CD3-targeting TCE blinatumomab, for B-cell precursor acute lymphoblastic leukaemia (ALL), demonstrated the clinical potential of this technology [2], setting off a wave of research to extend TCE therapies beyond haematologic malignancies and into solid tumours [3]. TCEs are typically engineered to bind tumour-associated antigens (TAAs) on cancer cells and the CD3 receptor on T cells, forming an immunological synapse that drives directed cytotoxicity [4].

The field has witnessed rapid technological advancement from simple bispecific formats to multispecific constructs that address key limitations of early-generation TCEs. The evolution from 1+1 bispecific formats to 2+1 configurations that harness avidity for improved tumour selectivity, and further to 1+1+1 trispecific designs that can simultaneously target multiple antigens or provide co-stimulatory signals, represents a systematic approach to overcoming therapeutic challenges. Latest developments in TCE technologies have also expanded their potential applications to solid tumours. For instance, targeting B7-H3, an immune checkpoint molecule associated with poor prognosis in several cancers, has been shown to augment the antitumor response of cytotoxic lymphocytes when engaged by TCEs or chimeric antigen receptor (CAR) T cells [5, 6]. TCEs are not only changing the treatment landscape of adult cancers, they are also significantly improving disease-free survival (DFS) rates in paediatric patients. In a recent Phase 3 clinical trial, adding blinatumomab to standard chemotherapy significantly improved DFS in paediatric patients with newly diagnosed B-cell acute lymphoblastic leukaemia (B-ALL). These current updates have the potential to establish a new standard of care practice [7].

The development of LAVA-1207, a bispecific gamma-delta TCE (Gammabody), for metastatic castration-resistant prostate cancer exemplifies the innovation in TCE design. LAVA-1207 targets the Vδ2 chain of Vγ9Vδ2-T cells and prostate-specific membrane antigen (PSMA) on prostate cancer cells, inducing potent T cell-mediated lysis of cancer cells while sparing normal cells [6]. This specificity and the anticipated low risk for cytokine release syndrome (CRS) highlight the potential of TCEs to provide effective and safer treatment options for patients [8].

However, the therapeutic value of TCEs is not without limitations. The transient nature of their efficacy, the need for continuous infusion, and the risk of CRS highlight the need for innovative solutions to enhance their clinical utility. But these challenges are also attributed to the other cellular therapy modalities in general [9]. Recent advancements in the field have focused on addressing these challenges through the development of half-life extended TCEs and combination therapies to mitigate toxicity while augmenting antitumor efficacy [10, 11].

As the field continues to mature, TCEs are positioned to play an increasingly central role in precision medicine approaches to cancer treatment and beyond. The combination of advanced engineering strategies, expanded target repertoires, and AI-driven optimisation promises to deliver next-generation TCEs with improved efficacy, safety, and applicability beyond oncology. This review examines the current state of TCEs, from fundamental mechanisms of action through clinical applications and manufacturing considerations, while highlighting emerging innovations that will shape the future of this transformative therapeutic modality.

## Mechanism of action and different T cell engager formats

### 1+1 format: foundation of bispecific design

**Fig. 1:**
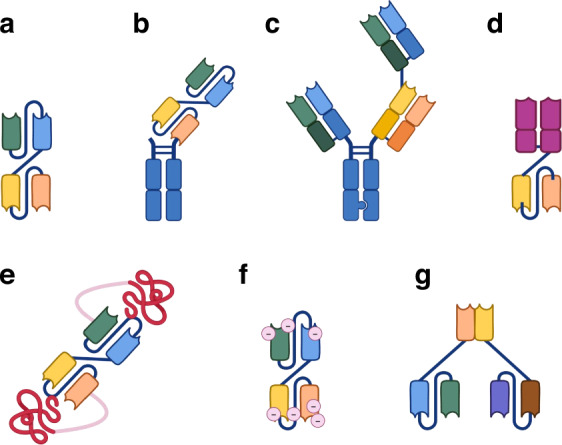
Major conventional and conditionally active T cell engager formats. **a** 1+1 format comprising two single-chain variable fragments (scFvs) connected by a flexible linker. **b** Half-life extended 1+1 format incorporating an Fc region to prolong systemic circulation. **c** 2+1 format containing two tumour-binding domains and one T cell-binding domain. Alternatively, this structure could be trispecific, with each domain recognising a distinct antigen. **d** TCE targeting intracellular antigens, consisting of an anti-CD3 arm fused to a T cell receptor (TCR)-based domain. **e** Protease-activated TCE in which a masking peptide containing protease cleavage sites conceals the antigen-binding domain, enabling conditional activation. **f** pH-sensitive TCE with the antigen-binding domain capped by pH-responsive physiological chemicals, allowing selective activation in the acidic tumour microenvironment. **g** Split TCE design where the variable light (VL) and variable heavy (VH) chains of the anti-CD3 domain are separated and fused to two distinct tumour-targeting antibodies, requiring co-localisation for full activity.

The 1+1 format refers to a TCE with one binding arm for a TAA and one for a T cell antigen (typically CD3). By simultaneously binding a cancer cell and a T cell, a 1+1 TCE creates a pseudo-immunological synapse that brings the T cell into close proximity with the tumour cell, triggering T cell activation and directed cytotoxicity. Upon engagement, the T cell releases perforin and granzymes to kill the tumour cell and secretes cytokines to amplify the immune response. A landmark example of the 1+1 format is blinatumomab, the first Food and Drug Administration (FDA)-approved TCE that targets CD19 in refractory B-ALL [12]. Notably, blinatumomab is a small single-chain TCE (~55 kDa) composed of two scFv (single-chain variable fragment) fragments (one anti-CD19, one anti-CD3) connected by a flexible linker (Fig. 1) [13].

Many recent 1+1 TCEs, such as mosunetuzumab and epcoritamab for CD20-positive lymphomas, as well as teclistamab and talquetamab for multiple myeloma, are built on an IgG backbone that includes an Fc domain. The Fc domain engages FcRn recycling pathways to greatly prolong circulation time, enabling intermittent dosing rather than continuous infusion [14]. Importantly, these Fc-containing TCEs are typically engineered with Fc mutations or IgG4 isotypes to abrogate Fcγ receptor and complement binding, thereby preventing off-target immune activation [15]. Despite their success, the efficacy of 1+1 TCEs is largely dependent on the presence of a single target antigen on cancer cells. Antigen loss has been reported in the relapse after treatment with TCE or CAR-T cells targeting CD19 [16].

Additionally, unlike a conventional bivalent antibody, a 1+1 TCE binds the target antigen monovalently, forfeiting the avidity advantage of dual-antigen binding. In a conventional IgG, even moderate-affinity binding can be converted into high-avidity attachment if the antigen is present in high density. This avidity helps discriminate cells with high antigen expression from those with low expression. As a result, 1+1 TCE targeting often relies on high-affinity binding, which can paradoxically reduce specificity by targeting cells with low antigen expression. For example, an anti-HER2×CD3 BiTE had to use an extremely high-affinity HER2 binder to be effective, but this risked recognising low-HER2 normal cells [17]. Thus, the monovalent nature of 1+1 TCEs presents a trade-off between potency and specificity. Newer designs like 2+1 formats (discussed next) and dual-antigen targeting trispecific TCE aim to overcome these issues by restoring avidity or requiring two antigens for activation.

### 2+1 format: harnessing avidity for selectivity

The 2+1 TCE format comprises two binding domains for the tumour antigen and one binding domain for CD3. By engaging two antigen molecules on the same cell, a 2+1 TCE achieves stronger binding to cells with high antigen density, while cells with low antigen density are bound weakly, reducing off-tumour effect. This ‘affinity tuning by avidity’ approach allows the use of lower-affinity tumour-binding domains that, on their own, might not bind to the antigen tightly, but in tandem yield avidity binding where there is a high expression of tumour antigen. As a result, 2+1 TCEs can better discriminate tumour cells from normal cells that express the antigens at lower levels. This strategy has proved effective with an anti-HER2×CD3 TCE, where TCE with two low-affinity anti-HER2 domains selectively eliminated HER2-overexpressing cancer cells while sparing cells with physiological HER2 levels [18].

Several 2+1 TCEs have advanced in the clinic, including the recently approved glofitamab (CD20×CD3) for relapsed/refractory diffuse large B-cell lymphoma. Glofitamab has two Fab arms targeting CD20 and a single arm targeting CD3, arranged in an IgG-like heterodimer [13]. In a Phase III trial of lymphoma, patients who received glofitamab plus gemcitabine–oxaliplatin achieved a better median overall survival of 25.5 months as compared to 12.9 months in patients who received rituximab plus gemcitabine–oxaliplatin [19]. Beyond CD20, the 2+1 format is being explored for other tumour antigens where avidity might be beneficial.

It’s important to clarify the terminology 2+1 vs 1+2 in TCE design. By convention, ‘2+1’ or ‘2:1’ refers to two tumour-binding units and one CD3-binding unit. The inverse 1:2 format, referring to one tumour binder and two CD3 binders, is generally avoided in practice, because a bivalent CD3 engagement might crosslink or activate T cells without a tumour present. Thus, most TCEs use a single CD3-binding arm. The 2+1 format remains the preferred way to harness avidity by increasing the selectivity towards tumour cells without increasing the risk of nonspecific T cell crosslinking.

### 1+1+1 trispecific formats: addressing complexity and resistance

Trispecific TCEs (1+1+1 format) contain three distinct binding domains, enabling recognition of three different antigens simultaneously. In the context of TCEs, one of these is typically CD3, and the other two are either two different tumour antigens or one tumour antigen plus an additional T cell co-stimulatory receptor. This format represents an advancement in traditional bispecifics, offering solutions to several challenges such as tumour antigen heterogeneity and insufficient T cell activation. There are two main classes of trispecific TCE designs: dual-tumour targeting and co-stimulatory TCEs.

### Dual-tumour targeting trispecific TCE

In this configuration, the TCE targets two different tumour antigens plus CD3. The rationale is to improve recognition of heterogeneous tumours and prevent antigen escape. A cancer cell that loses one antigen may still express the second, allowing the TCE to bind and kill it via the remaining arm. Conversely, if a tumour cell expresses both target antigens, the TCE can bind both simultaneously, achieving a higher avidity binding to that cell. For example, ISB 2001 is a trispecific TCE that targets BCMA and CD38 on multiple myeloma cells, plus CD3 [20]. In preclinical studies, ISB 2001 was able to robustly kill myeloma cells even if one of the antigens (BCMA or CD38) was absent. If both antigens were present, the dual engagement yielded superior tumour cell killing. Another dual-target example is a CD19×CD22×CD3 TCE reported for B-ALL [20]. By targeting two distinct B-lineage antigens (CD19 and CD22), it delayed antigen-loss mediated relapse in mouse models and improved overall leukaemia clearance. These findings underscore that 1+1+1 dual-target TCEs can address tumour heterogeneity and reduce the likelihood of immune escape.

### Co-stimulatory trispecific TCE

The second major category of trispecific TCE involves targeting an additional T cell receptor (TCR), typically a co-stimulatory molecule, alongside CD3 and a tumour antigen. This design directly tackles the issue that T cells require not just TCR signalling (often called ‘Signal 1’) but also a co-stimulatory signal (‘Signal 2’, such as via CD28) for full activation, proliferation, and sustained killing function [21]. In tumours, especially solid tumours with immunosuppressive environments, T cells may receive suboptimal co-stimulation, leading to T cell exhaustion or anergy. A trispecific TCE with a co-stimulatory arm can provide that missing second signal, with CD28 being one of the most common choices [22]. One example is a TCE targeting CD3, CD28, and PSMA (prostate-specific membrane antigen) in prostate cancer [23]. It triggered greater T cell activation, cytokine production (like IL-2 and TNF-α), and the formation of effector memory T cells than the CD3xPSMA TCE. In addition to CD28, other co-stimulatory or immune-modulatory receptors can be targeted. For instance, a similar observation in immune response as of CD28 was also observed when the co-stimulatory domain 4-1BB was being targeted [23]. Interestingly, when OX40 was targeted, the TCE triggered the expansion of CD4^+^ central memory T cells with fewer Tregs induction, highlighting the importance of the selection of co-stimulatory molecules to be targeted. While co-stimulatory trispecifics are still in early-phase trials, they represent a promising strategy to improve T cell potency and persistence, enabling better outcomes in solid tumours where T cells are often immunosuppressed.

### TCEs targeting intracellular antigens through HLA presentation/ expanding the target antigen universe

Conventional TCEs target antigens present on the cell surface of tumour cells. However, many tumour-specific antigens, such as oncoproteins and viral antigens, are intracellular proteins that are not accessible to conventional TCE. To redirect T cells against intracellular antigens, newer TCE modalities employ binding domains that recognise peptide–HLA complexes. In essence, they combine the specificity of TCRs with the TCE principle of CD3 engagement.

A great example is tebentafusp, the first TCR-based TCE approved for uveal melanoma [24]. It contains a high-affinity TCR specific for a peptide from the gp100 melanoma antigen presented by HLA-A02:01, fused to an anti-CD3 scFv [25]. This molecule binds to melanoma cells only if they present the gp100 peptide on the HLA-A2 molecule. In a randomised trial in metastatic uveal melanoma, tebentafusp significantly extended median overall survival to 21.6 months as compared to 16.9 months in the control group (pembrolizumab, ipilimumab, or dacarbazine), validating the approach of targeting peptide–HLA complexes [24].

Beyond tebentafusp, a number of TCR-mimicking TCEs are in development. These use either engineered human TCRs or antibody fragments that recognise a specific peptide–HLA complex. For example, a bispecific TCE targeting an intracellular oncogene WT1 was created using a TCR-like antibody that binds the WT1 peptide/HLA-A02:01 complex [26]. Similarly, high-affinity soluble TCRs have been identified for mutant neoantigens like KRAS^G12D^, a common mutation in pancreatic and colorectal cancers [27]. A study described a TCR-based biologic that targeted the KRAS^G12D^ peptide presented by HLA-A11:01, showing potent killing of KRAS-mutant tumour cells by T cells [28]. Research is also underway against the p53 hotspot mutation R175H. A recent report detailed a TCE that bound the HLA-A*02:01 complex of p53^R175H^ peptide and CD3 to selectively lyse p53-mutant tumour cells in vitro [29]. These examples highlight the expanding repertoire of intracellular targets being brought into reach by TCE technology.

The major limitation of targeting peptide–HLA complexes is the HLA restriction. Human populations are diverse in HLA types, so a peptide that is presented by one allele, such as HLA-A*02:01, might not be presented in another patient who lacks that allele. This means such TCEs need to be developed in an allele-specific manner. Nonetheless, this strategy opens up a vast pool of cancer antigens, including oncogenes, tumour suppressors, cancer-testis antigens, and viral antigens in virus-driven cancers, that were previously ‘undruggable’ by antibodies.

## Overcoming key therapeutic challenges

### Cytokine release syndrome (CRS): mechanisms and mitigation

CRS is an acute toxicity that results from the rapid and massive release of inflammatory cytokines by T cells and other immune cells [30]. Clinically, CRS presents with fever, hypotension, tachycardia, and in severe cases can progress to capillary leak, multi-organ dysfunction [30]. In patients receiving TCEs, activated T cells secrete IFNγ, TNFα, and interleukins, such as IL-2, IL-6, which trigger a systemic inflammatory cascade. Monocytes and macrophages that are often activated secondarily, for instance by IFNγ from T cells, are major sources of IL-6 and IL-1 that drive many CRS symptoms [31]. The incidence of CRS varies with the TCE. For instance, trials of blinatumomab reported CRS in ~11–15% of patients, with severe (grade ≥ 3) CRS in 2–5% [32]. Mechanistically, TCEs can polyclonally activate any T cell regardless of its native antigen specificity, as long as the target antigen is present, leading to an army of T cells releasing cytokines.

It has been observed that T cell killing can occur with a relatively low-intensity CD3/TCR engagement, whereas robust cytokine secretion (and full T cell activation) requires a higher antigen intensity [33]. In other words, there appears to be a dual threshold in T cell activation: one threshold for cytotoxicity and a higher threshold for cytokine release. TCEs that strongly engage CD3 can drive T cells above both thresholds, resulting in both target cell lysis and massive cytokine production. The cytokines, IL-6 in particular, then mediate the systemic symptoms of CRS.

To mitigate CRS, several strategies have been employed. Clinically, step-up dosing is commonly used, in which patients receive a very low first dose of the TCE, then gradually escalating doses on subsequent days, allowing the immune system to acclimate [34]. The initial T cell activation at a low dose might cause some cytokine release and T cell expansion. However, because the dose is low, the CRS is milder, resulting in the gradual priming of the immune system to prevent an early, uncontrolled inflammatory response [35]. This approach significantly lowered the onset and severity of CRS rates associated with TCE therapy. Prophylactic or early intervention with immunosuppressive drugs is another strategy. For example, administering corticosteroids or an IL-6 receptor blocker (tocilizumab) at the first sign of CRS can minimise the likelihood of the severe grades of adverse events [36].

On the drug design side, a major approach to reduce CRS without sacrificing anti-tumour activity has been tuning of the affinity of the CD3-binding domain. By reducing the affinity, the TCE induces a less vigorous T cell activation signal, staying below the cytokine-release threshold while still triggering cytotoxicity. Preclinical studies demonstrated that lowering CD3 affinity can uncouple cytotoxic efficacy from cytokine secretion, where T cells still killed target cells efficiently, but produced significantly less IL-2 and IL-6 [37, 38].

### Pharmacokinetics and half-life extension strategies

Another challenge for early TCE therapies was their short plasma half-life. TCEs without the Fc domain are rapidly cleared by renal filtration and lack mechanisms for recycling in the body. For example, blinatumomab has a half-life is ~2 h, which thereby requires continuous intravenous infusion over 4 weeks of a 6-week cycle [39]. This is logistically inconvenient for patients and healthcare systems. Prolonging the half-life of TCEs has therefore been a focus in molecular design to enable convenient dosing schedules while maintaining steady exposure to the therapeutics.

The primary strategy for half-life extension has been to incorporate an Fc domain into the TCE construct. The Fc region confers two advantages by increasing the molecular size to mitigate renal clearance and by binding to the neonatal Fc receptor (FcRn), which salvages the antibody from lysosomal degradation [14]. They achieve half-lives of 5–7 days, allowing dosing intervals of once every week or two. Most of the Fcs are engineered to be ‘silenced’ to abort any interaction with the Fcγ receptor or complement pathways [15].

Another half-life extension approach is via albumin binding. Human serum albumin is an abundant ~67 kDa serum protein with a long half-life of ~20 days due to the FcRn-mediated recycling, similar to IgG [40]. By attaching an albumin-binding domain to a TCE, the drug can bind to albumin to undergo FcRn recycling. Anti-albumin single-domain antibody has been used to build TCE in one single-chain protein. The albumin-binding confers a long serum half-life (on the order of days) despite the relatively small size of the trispecific TCE (~53 kDa) [41].

A more fine-tuned method of adjusting how long the TCE stays in circulation is by altering the binding affinity of the Fc or albumin-binding domain to FcRn. By increasing FcRn binding, one can further extend half-life [42]. Conversely, slightly decreasing FcRn binding can shorten half-life if a very prolonged exposure is not desired for safety. It was reported that by modulating FcRn interaction, bispecific TCE-albumin fusion proteins achieved a ‘programmable’ half-life between ~19 h and ~37 h in murine as compared to 0.6 h from the TCE that were not fused to the albumin [43].

### Minimising off-tumour effects through conditional activation

A significant concern for TCE therapy is on-target, off-tumour toxicity. Unlike the haematologic antigens CD19 or BCMA, which are mostly restricted to B-lineage cells or plasma cells, solid tumour antigens are often shared with some healthy tissues. When a TCE directs T cells to any cell expressing the target antigen, it does not inherently distinguish a cancer cell from a normal cell if the antigen is present on both, resulting in serious off-tumour toxicities. To address this, researchers have designed conditionally active TCEs, which remain inert in normal tissues and become activated only in the tumour microenvironment. These act as prodrugs that require a tumour-specific trigger to unleash their T cell-engaging activity. Several strategies have been developed:

#### Protease-activated TCEs

Tumour-associated proteases, enzymes that are abundantly expressed in the tumour microenvironment, are often used as the trigger [44]. The TCE is built with a masking peptide covering the key binding domain, usually the anti-CD3 arm, via a linker that contains protease cleavage sites [45, 46]. In circulation and normal tissues, the mask sterically blocks the CD3-binding site, preventing the TCE from engaging T cells. However, in the tumour microenvironment, where proteases like matrix metalloproteinases are abundant, the linker is cleaved, shedding the mask and exposing the CD3-binding domain, allowing the engagement of the tumour-infiltrating T cells. A notable example of this approach is a masked EGFRxCD3 TCE that is currently in Phase I trials for EGFR-expressing solid tumours [47]. Other protease-activated TCEs are being developed for different tumour targets, such as B7-H3, based on the principle of exploiting the biochemical differences of the tumour microenvironment, to activate the drug at the cancer site [48].

#### pH-sensitive TCEs

Another distinguishing feature of the tumour microenvironments is acidosis, as rapidly growing tumours often have poor perfusion and rely on anaerobic glycolysis [49], leading to lactic acid accumulation and an extracellular pH that can be as low as 6.7–7.1 (versus ~7.4 in normal tissue). pH-sensitive TCEs are engineered to bind their targets only at acidic pH and not at physiological pH. One innovative technique involves the generation of an antibody mutant library to identify variants that demonstrate conditional binding at low pH [50]. At neutral pH, the selected variant remains bound by physiological chemicals like bicarbonate or hydrogen sulphide, and is effectively ‘capped’ by these chemical plugs. When the TCE diffuses into acidic tumour tissue, the excess hydrogen ions protonate and dissociate the blocking chemicals from the binding site, thereby the TCE becomes free to bind the target antigen and CD3. A recent study applied this concept to create a pH-conditional anti-EpCAM×CD3 bispecific antibody [51]. The TCE showed minimal binding to EpCAM at pH 7.4 but robust binding at pH 6.0, triggering preferential killing at acidic pH. This approach is still in preclinical or early development, but holds promise for improving the tumour specificity of TCE therapy.

#### Split TCE systems

Although not yet in the clinic, some groups have proposed splitting the TCE into two halves that must come together for activity. For example, one half of the bispecific could contain the tumour-binding domain and the variable heavy chain (VH) of an anti-CD3 arm, and the other half contains another tumour-binding arm and the variable light chain (VL) of the same anti-CD3 arm [52]. Each half by itself is inactive and cannot bridge the T cell to the cancer cell. If both halves bind to the cancer cell, their VH and VL of CD3 domains heterodimerise, reconstituting a functional CD3-binding domain for T cell engagement.

### Production methodologies and manufacturing challenges

Manufacturing TCE presents significant challenges due to its bivalency and unconventional antibody structure. Key considerations include achieving proper pairing of multiple antibody chains, maintaining protein purity, and obtaining sufficient yield for clinical use.

#### Ensuring correct chain assembly in complex constructs

A central challenge in producing IgG-like TCEs is that they typically involve four polypeptide chains (two heavy and two light chains) from two different antibodies. If all are expressed together, there are many possible pairings, potentially leading to mispaired heavy-heavy homodimers or heavy-light mismatches. To address this, several engineering strategies have been developed.

##### Knob-into-Hole (KiH) mutations

This is a genetic engineering method that promotes the heterodimerisation of two different heavy chains. The CH3 region of one heavy chain is mutated to have a bulky hydrophobic ‘knob’, while the other CH3 is mutated to have a complementary ‘hole’ [53–55]. When co-expressed, the knobbed heavy chain preferentially pairs with the hole-containing heavy chain, rather than with itself, thus favouring the formation of the desired heterodimer and bispecific structure.

##### CrossMab technology

Knob-into-hole fixes the heavy-heavy pairing, but does not by itself ensure that each heavy chain finds its correct light chain. CrossMab approach solves this by swapping domains between heavy and light chains for one of the Fab arms [56, 57]. For example, one approach is to exchange the VH and CH1 domains of one antibody with its corresponding light-chain domains (VL and CL). This way, the light chain will only correctly pair with the heavy chain that has the complementary swapped domain.

##### Common light chain

Another strategy is to design the two different antigen-binding Fabs of a bispecific to share the same light-chain sequence [58, 59]. With a common light chain, there is no mismatched light chain for a given heavy chain. The heavy-heavy heterodimer can be enforced by KiH.

##### Fab-arm exchange (FAE)

In vitro assembly after antibody production via controlled Fab-arm exchange is another possible strategy. In this approach, two parental IgG antibodies are mixed under conditions that transiently reduce the disulphide bonds to promote half-molecule exchange, and the pairing process is further enhanced by the mutations in the CH3 region [60].

#### Expression platform selection and optimisation

Most clinical TCEs today are produced in mammalian cell systems (primarily Chinese hamster ovary, CHO, cells) because these systems provide correct protein folding, assembly, and post-translational modifications [61, 62]. Meanwhile, transient transfection of HEK293 or CHO cells is often used in early development to rapidly produce TCE candidates.

For certain smaller TCE formats, especially those composed solely of linked fragments, microbial expression systems such as E. coli can achieve very high expression of recombinant protein (multi-gram per litre) [63]. However, proteins expressed in E. coli often accumulate in inclusion bodies as insoluble aggregates [64]. This requires additional denaturing and in vitro refolding of the protein to retrieve a functional product.

Other hosts, like yeast, have also been explored [65]. Yeast, being a eukaryote, can perform post-translational modification and often secrete folded proteins, while growing faster than most mammalian cells [66, 67]. Additionally, cell-free expression systems are another emerging method [68]. While cell-free manufacturing offers speed and precise control, it currently has higher costs and scalability limitations. Expression in mammalian cells remains the dominant approach for commercial TCE production.

## Clinical landscape and approved T cell engagers

### First-generation success

The first approved TCE was catumaxomab; it engages EpCAM-positive tumour cells and T cells, inducing T cell-mediated cancer cell killing. Catumaxomab was the first-generation TCE that has a fully functional Fc region of catumaxomab binds to Fc receptors (FcRs) on macrophages and NK cells [69]. First attempts for intravenous delivery failed due to severe liver toxicity that is linked to Fc-mediated off-target Kupffer and T cell activation in the liver [70]. Then it was approved in the European Union in 2009 for the intraperitoneal treatment of malignant ascites in patients with EpCAM-positive carcinomas, but catumaxomab was not well tolerated intravenously as there was nonspecific activation of T cells due to CRS. Therefore, it was withdrawn by the FDA in 2013 and then by the European Medicines Agency (EMA) in 2017 for commercial reasons [71]. Next-generation TCE designs include inert constant regions that can both minimise Fc-mediated T cell toxicity and enhance lymphocyte trafficking and anti-tumour potency [70]. Then it was a while until, in 2014, Blinatumomab was granted accelerated approval for the treatment of precursor B-ALL [12].

The CD19/CD3 BiTE molecule, blinatumomab, has demonstrated the clear proof of concept of antitumor activity and clinical efficacy of TCEs in treating B-cell malignancies. Its proven clinical efficacy and favourable safety profile resulted in it becoming the first of its kind to be approved by both the EMA and the U.S. FDA for use in both children and adults with relapsed or refractory Philadelphia chromosome (Ph)-negative precursor B-ALL [72].

Currently, FDA-approved TCEs are used to treat patients with certain blood cancers multiple myeloma, leukaemia, lymphoma, small cell lung cancer and melanoma. More recent FDA-approved TCEs include glofitamab (CD20 × CD3), mosunetuzumab (CD20 × CD3), teclistamab (BCMA × CD3), talquetamab (GPRC5D × CD3), indicating the expanding scope of TCEs in haematologic malignancies and solid tumours (Table 1) [73].

**Table 1:** Approved TCEs for cancer.

TCE (trade name)	Disease indication	Mechanism of action
Blinatumomab (Blincyto)	Acute lymphocytic leukaemia (ALL)	Blinatumomab links T cells with CD19 receptors found on the surface of B cells.
Glofitamab (Columvi)	Elapsed or refractory diffuse large B-cell lymphoma, not otherwise specified (DLBCL, NOS) or large B-cell lymphoma (LBCL).	Glofitamab binds the CD3 receptor in T cells and links them to CD20 surface antigens found on B cells.
Mosunetuzumab (Lunsumio)	Follicular lymphoma.	Mosunetuzumab binds the CD3 receptor in T cells and links them to CD20 surface antigens found on B cells.
Tarlatamab (Imdelltra)	Small cell lung cancer (SCLC)	Binds to DLL3 on tumour cells and CD3 on T cells, tarlatamab enables the recruitment and activation of T cells.
Tebentafusp (Kimmtrak)	Unresectable or metastatic uveal melanoma	Binds gp100 (a melanoma-associated antigen) through a high-affinity T cell receptor (TCR) binding domain and an anti-CD3 T-cell-engaging domain
Elranatamab (Elrexfio™)	Multiple Myeloma (MM)	BCMA on MM and CD3 on T cells.
Epcoritamab (Eplinky)	Diffuse large B-cell lymphoma	CD3xCD20 T cell–engaging, bispecific antibody that activates T cells, directing them to kill malignant CD20+ B cells.
Teclistamab (Tecvayli)	Multiple Myeloma (MM)	Targeting both the CD3 receptor complex on T cells and BCMA on myeloma cells, teclistamab leads to T cell activation and subsequent lysis of BCMA+ cells.
Talquetamab (Talvey)	Multiple Myeloma	It is a bispecific GPRC5D-directed CD3 T cell engager.

*MM* multiple myeloma, *BCMA* B-cell maturation antigen, *TCR* T cell receptor.

### Target profiles and novel targets

TCEs usually target extracellular proteins, and the ideal target for TCEs should be expressed on the tumour cell surface, whereas not on healthy cells, to prevent on-target off-tumour toxicities. As target antigen loss and downregulation are one of the most common evasion mechanisms in cancer, targeted antigen should also play a major role in tumour cell survival [74].

Rapid target internalisation can negatively impact the activity of the TCE, as this will limit immune synapse formation, as only cell surface-bound TCEs can form an immune synapse. New TCE formats have been developed to detect peptides from intracellular proteins presented by HLA molecules on the cell surface. These formats are particularly valuable because most TAAs originate from intracellular proteins. By systematically profiling the surfaceome of cancer cells, novel TAAs can be identified. These new TCE formats target various aberrantly expressed intracellular oncogenic antigens, TAAs, mutant oncogenes, and tumour suppressor genes [75].

**Fig. 2:**
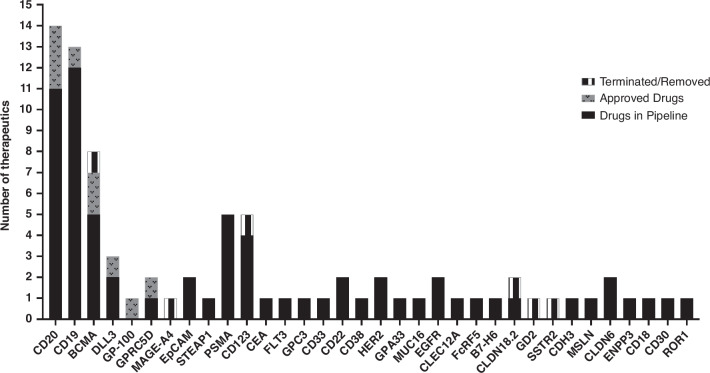
T cell engager target profiles in clinical development for cancer therapy. More than 200 TCE antibodies are currently in clinical trials. Approximately half of the bispecific antibodies are targeted to CD20, CD19 and BCMA. Nonetheless, newer targets have also entered early-phase clinical trials.

A key aspect of developing effective TCEs is the careful selection of targets. CD19 and CD20 are relatively stable cell-surface antigens widely expressed on B cells [76]. Therefore, most targeted surface antigens include CD19, CD20, and BCMA (B-cell maturation antigen) in ongoing clinical trials Table 2, Fig. 2. The development of TCEs targeting solid tumour antigens such as PSMA (prostate-specific membrane antigen), EpCAM (epithelial cell adhesion molecule), CEA (carcinoembryonic antigen), and GPC3 (glypican-3) marks a significant advancement in expanding this technology beyond haematological malignancies. These BiTEs are designed to address the unique challenges posed by solid tumours, which include a more complex and immunosuppressive tumour microenvironment compared to haematological cancers (Fig. 2).

**Table 2:** Beyond oncology.

Molecule	Disease indication	Targets	Phase
Mosunetuzumab (Lunsumio)	Systemic lupus erythematosus	CD20xCD3	Phase 1
Blinatumomab (Blincyto)	Refractory rheumatoid arthritis	CD19xCD3	N/A
IGM2644	Autoimmune disease	CD38xCD3	Preclinical

## Applications beyond oncology

Although haematologic malignancies have been the primary area of TCE success, new designs aim to overcome the immunosuppressive TME in solid tumours. Examples include TCEs targeting PSMA in prostate cancer and EGFR in non-small cell lung cancer. Innovative technologies, such as mask-and-unmask platforms or pH-dependent binding, seek to enhance tumour specificity and reduce systemic toxicities [77, 78].

TCEs have primarily been developed and utilised in oncology, particularly for haematologic malignancies and solid tumours. However, there is growing interest and potential for their application in non-oncology indications, such as autoimmune and infectious diseases [79, 80]. Some targets, like CD38, could have potential value for both oncology and non-oncology (systemic autoimmune diseases). CD38 is a multifunctional transmembrane glycoprotein that plays a significant role in both cancer and systemic autoimmune diseases. It is highly expressed in various tumour types, including multiple myeloma, leukaemia, and solid tumours. Its overexpression is linked to cancer progression, angiogenesis, and immune suppression, making it a promising target for therapeutic interventions. Monoclonal antibodies targeting CD38, such as daratumumab and isatuximab, have shown substantial success in treating multiple myeloma and are being explored for other malignancies [81–83].

In systemic autoimmune diseases, CD38 is expressed on various immune cells, including plasma cells, which are responsible for producing autoantibodies. Targeting CD38 with monoclonal antibodies like daratumumab has emerged as a promising therapeutic approach for autoimmune conditions such as systemic lupus erythematosus, rheumatoid arthritis, and ANCA-associated vasculitis. By depleting autoantibody-producing plasma cells and modulating immune cell functions, anti-CD38 therapy has the potential to reduce disease activity and prevent disease progression in these debilitating conditions. This dual role of CD38 in both oncology and immunology underscores its importance as a therapeutic target across a spectrum of diseases [81, 83, 84].

### Future directions and technological innovations

#### Artificial intelligence in TCE design

Artificial intelligence (AI) opened up a new era as a vital tool in accelerating drug design, particularly for complex constructs like TCEs. AI-driven methods can analyse vast datasets of antibodies, TCRs, and tumour antigens to optimise binding specificity, reduce immunogenicity, and minimise off-target effects [85].

By integrating AI-driven structure prediction (AlphaFold and Rosetta), generative design, and physics-based simulations, biotechnology companies are advancing TCEs at unprecedented speed and precision [86]. As these computational platforms continue to improve, there will be more specific TCEs for both cancer and non-oncology indications, addressing core challenges of toxicity, efficacy, and scalability in immunotherapy. Ongoing projects also include using a machine learning-driven approach to optimise TCEs for the treatment of advanced cancers, enabling candidates with better efficacy and safety to be taken forward for future development. LabGenius has demonstrated how Machine learning can systematically optimise multiple properties (e.g. selectivity, potency and developability) in TCE design. Their ML-driven pipeline delivered HER2×CD3 antibodies with over 400-fold improvement in tumour-selective killing compared to a clinical benchmark [87].

## Conclusion

TCEs have rapidly evolved from early-generation bispecific antibodies to multispecific constructs capable of targeting multiple tumour antigens or co-stimulatory pathways. Their success in haematologic malignancies and emerging promise in solid tumours demonstrates the promise of TCE-based immunotherapies. Ongoing research focuses on refining antigen selectivity, extending half-life, minimising CRS, and mitigating off-tumour toxicities. Furthermore, there is a growing interest in non-oncology applications, leveraging TCE-mediated cytotoxicity in autoimmune and infectious diseases. As newer formats and combination strategies emerge, TCEs are positioned to reshape cancer immunotherapy by offering hope for more precise, potent, and safer immune-mediated therapies.

TCEs have revolutionised immunotherapy by directing T cell cytotoxicity toward tumour cells, leading to significant clinical successes in haematologic malignancies and promising activities in solid tumours. Advances in TCE designs, including the 1+1, 2+1, and 1+1+1 formats, have enhanced target avidity, addressed tumour antigen heterogeneity, and improved safety profiles. Next-generation TCEs are tackling intracellular antigens via TCR-based mechanisms and are expanding beyond oncology into autoimmune and other disease indications. Challenges such as CRS, short half-lives, and off-tumour toxicity are being mitigated through innovative engineering strategies, while AI-driven methods offer unique opportunities to fine-tune TCE architecture. As TCE development progresses, its successful integration into combination therapies will likely determine the next wave of breakthroughs.
